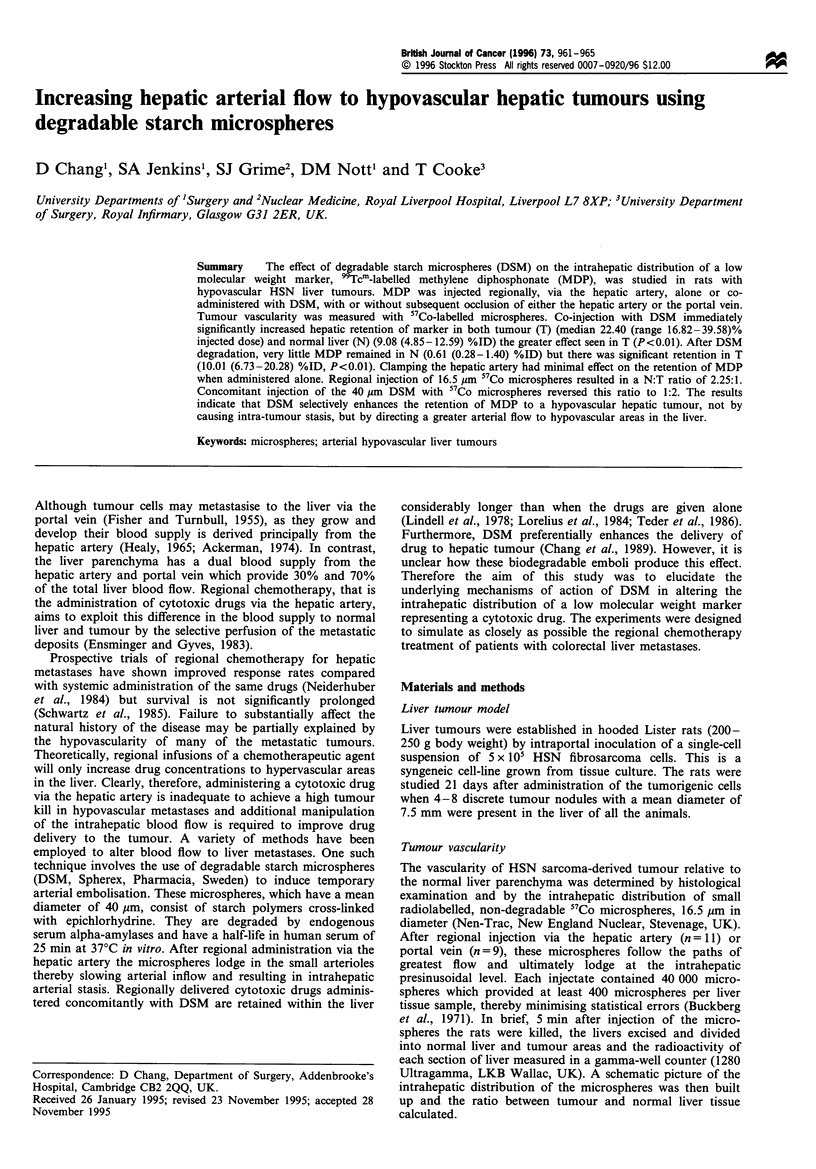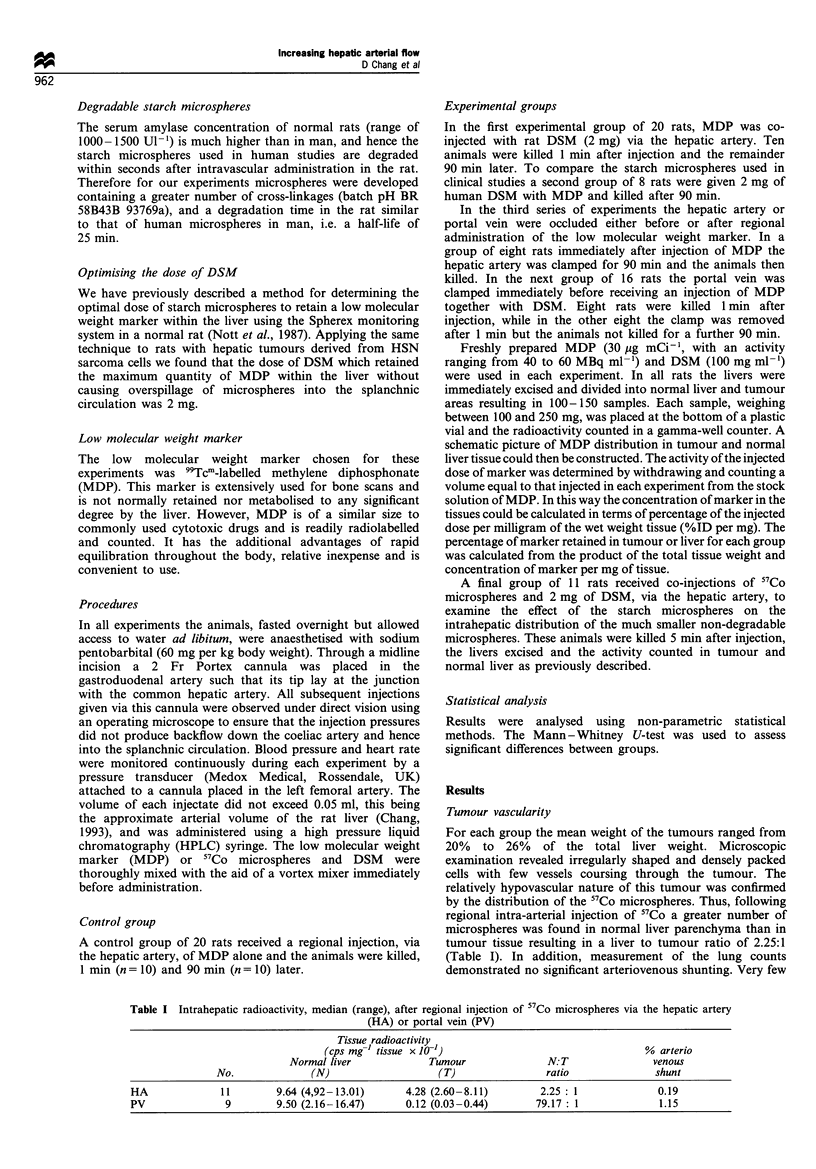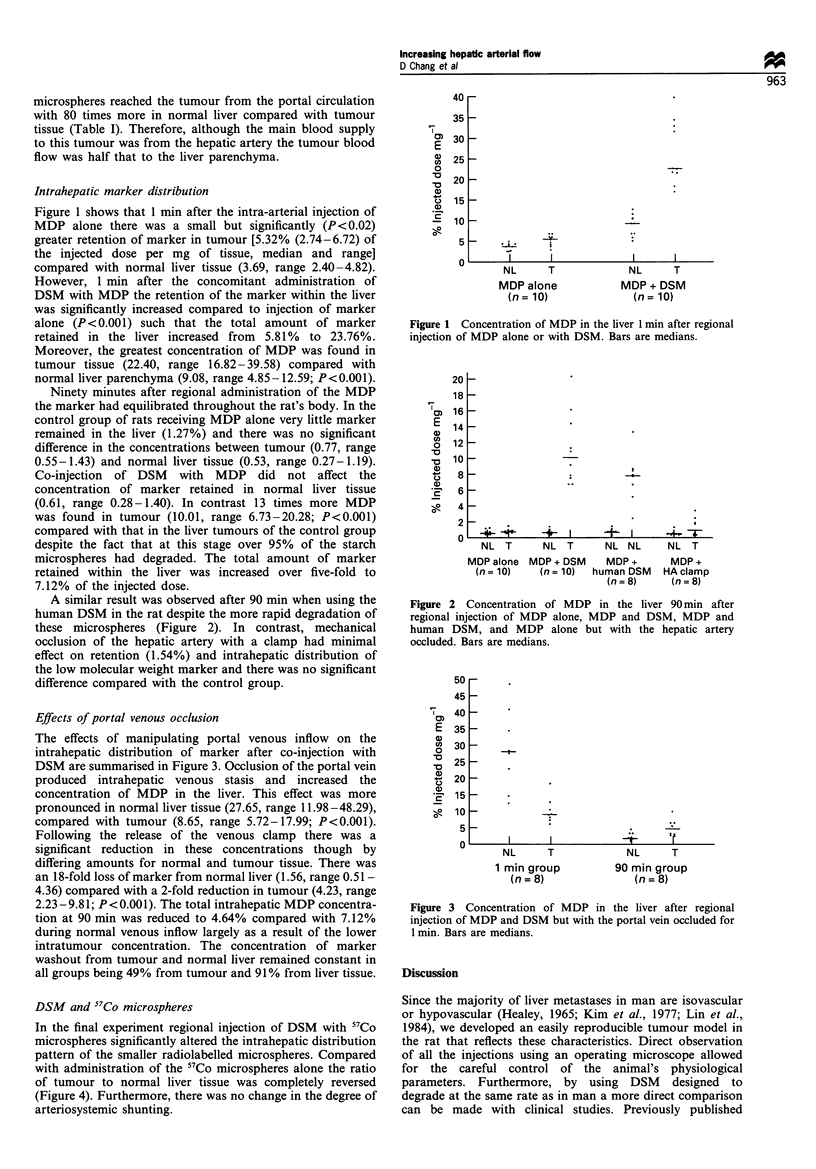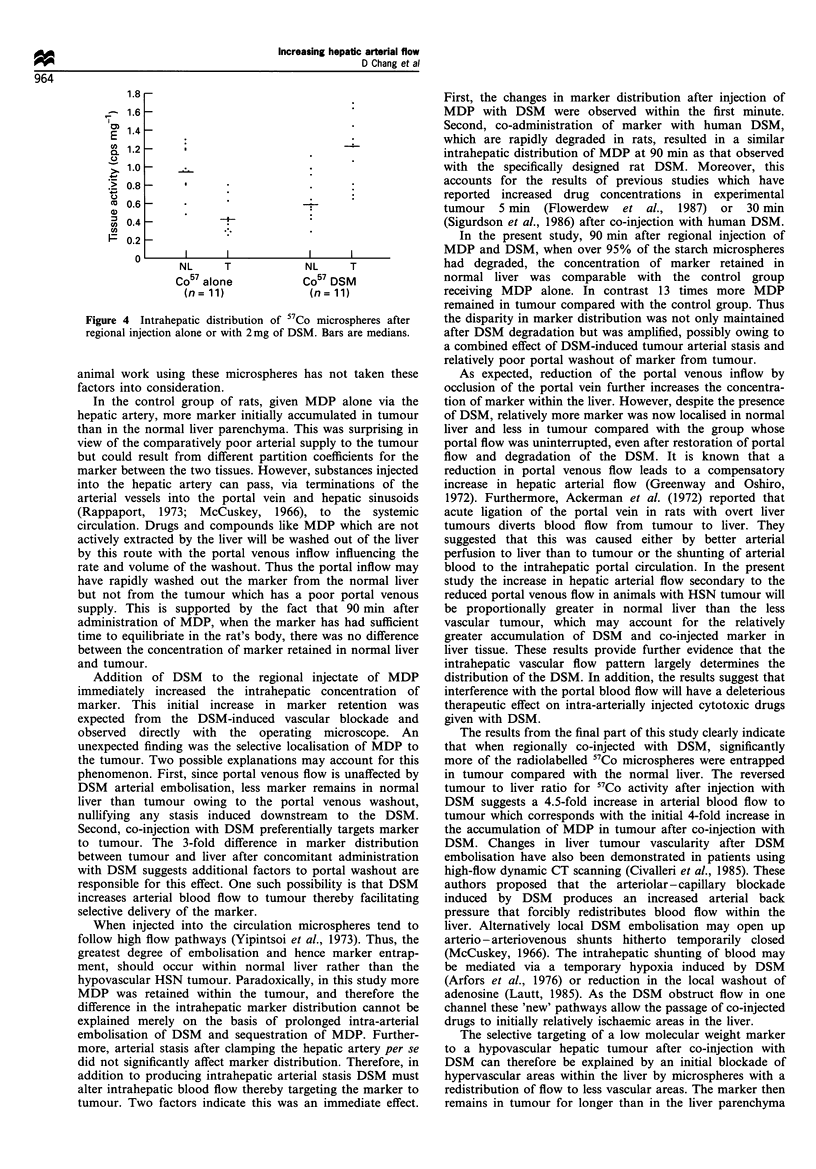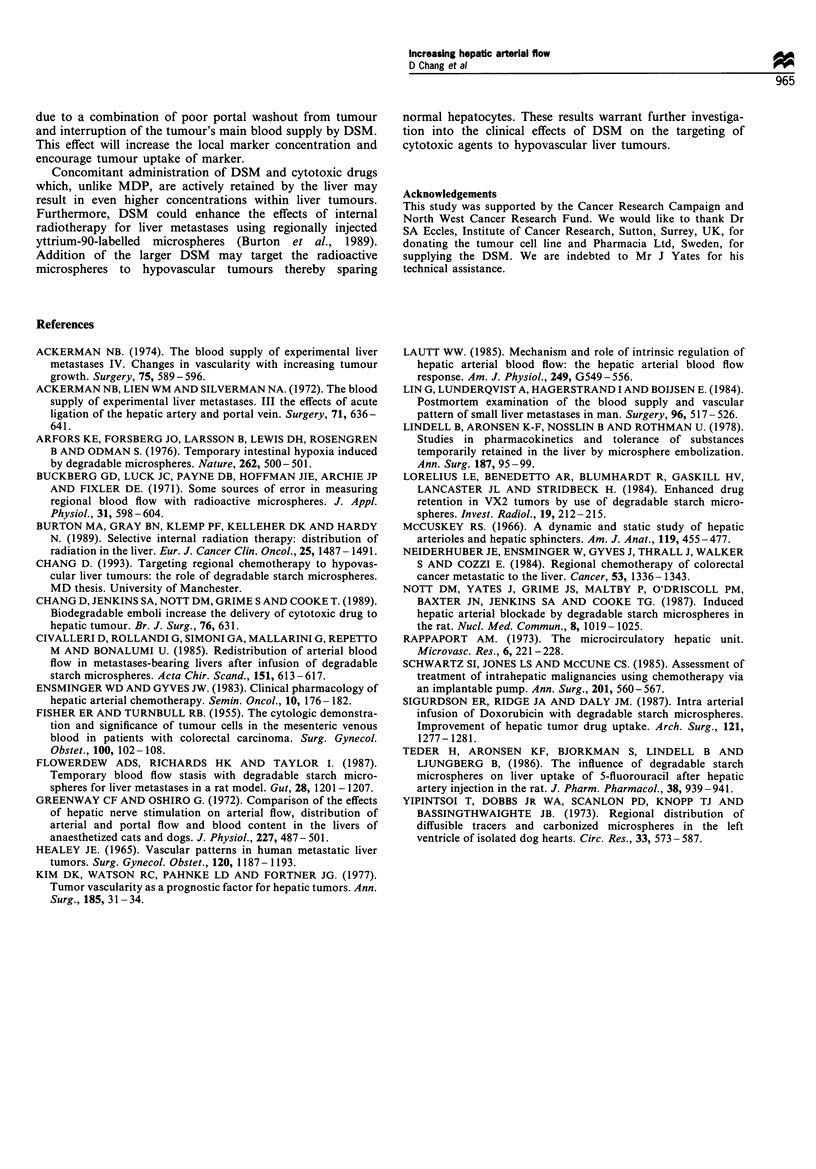# Increasing hepatic arterial flow to hypovascular hepatic tumours using degradable starch microspheres.

**DOI:** 10.1038/bjc.1996.188

**Published:** 1996-04

**Authors:** D. Chang, S. A. Jenkins, S. J. Grime, D. M. Nott, T. Cooke

**Affiliations:** University Departments of Surgery, Royal Liverpool Hospital, UK.

## Abstract

The effect of degradable starch microspheres (DSM) on the intrahepatic distribution of a low molecular weight marker, 99Tcm-labelled methylene diphosphonate (MDP), was studied in rats with hypovascular HSN liver tumours. MDP was injected regionally, via the hepatic artery, alone or co-administered with DSM, with or without subsequent occlusion of either the hepatic artery or the portal vein. Tumour vascularity was measured with 57Co-labelled microspheres. Co-injection with DSM immediately significantly increased hepatic retention of marker in both tumour (T) (median 22.40 (range 16.82-39.58)% injected dose) and normal liver (N) (9.08 (4.85-12.59) %ID) the greater effect seen in T (P < 0.01). After DSM degradation, very little MDP remained in N (0.61 (0.28-1.40) %ID) but there was significant retention in T (10.01 (6.73-20.28) %ID, P < 0.01). Clamping the hepatic artery had minimal effect on the retention of MDP when administered alone. Regional injection of 16.5 microM 57Co microspheres resulted in a N:T ratio of 2.25:1. Concomitant injection of the 40 microM DSM was 57Co microspheres reversed this ratio to 1:2. The results indicate that DSM selectively enhances the retention of MDP to a hypovascular hepatic tumour, not by causing intra-tumour stasis, but by directing a greater arterial flow to hypovascular areas in the liver.


British Journal of Cancer (1996) 73, 961-965
© 1996 Stockton Press All rights reserved 0007-0920/96 $12.00

### Increasing hepatic arterial flow to hypovascular hepatic tumours using degradable starch microspheres

D Chang[1], SA Jenkins[1], SJ Grime[2], DM Nott[1] and T Cooke[3]

*University Departments of [1]Surgery and [2]Nuclear Medicine, Royal Liverpool Hospital, Liverpool L7 8XP; [3]University Department of Surgery, Royal Infirmary, Glasgow G31 2ER, UK.*

**Summary** The effect of degradable starch microspheres (DSM) on the intrahepatic distribution of a low molecular weight marker, $^{99}Tc^m$-labelled methylene diphosphonate (MDP), was studied in rats with hypovascular HSN liver tumours. MDP was injected regionally, via the hepatic artery, alone or co-administered with DSM, with or without subsequent occlusion of either the hepatic artery or the portal vein. Tumour vascularity was measured with $^{57}Co$-labelled microspheres. Co-injection with DSM immediately significantly increased hepatic retention of marker in both tumour (T) (median 22.40 (range 16.82-39.58)% injected dose) and normal liver (N) (9.08 (4.85-12.59) %ID) the greater effect seen in T ($P<0.01$). After DSM degradation, very little MDP remained in N (0.61 (0.28-1.40) %ID) but there was significant retention in T (10.01 (6.73-20.28) %ID, $P<0.01$). Clamping the hepatic artery had minimal effect on the retention of MDP when administered alone. Regional injection of 16.5 $\mu m$ $^{57}Co$ microspheres resulted in a N:T ratio of 2.25:1. Concomitant injection of the 40 $\mu m$ DSM with $^{57}Co$ microspheres reversed this ratio to 1:2. The results indicate that DSM selectively enhances the retention of MDP to a hypovascular hepatic tumour, not by causing intra-tumour stasis, but by directing a greater arterial flow to hypovascular areas in the liver.

**Keywords:** microspheres; arterial hypovascular liver tumours

Although tumour cells may metastasise to the liver via the portal vein (Fisher and Turnbull, 1955), as they grow and develop their blood supply is derived principally from the hepatic artery (Healy, 1965; Ackerman, 1974). In contrast, the liver parenchyma has a dual blood supply from the hepatic artery and portal vein which provide 30% and 70% of the total liver blood flow. Regional chemotherapy, that is the administration of cytotoxic drugs via the hepatic artery, aims to exploit this difference in the blood supply to normal liver and tumour by the selective perfusion of the metastatic deposits (Ensminger and Gyves, 1983).

Prospective trials of regional chemotherapy for hepatic metastases have shown improved response rates compared with systemic administration of the same drugs (Neiderhuber *et al.*, 1984) but survival is not significantly prolonged (Schwartz *et al.*, 1985). Failure to substantially affect the natural history of the disease may be partially explained by the hypovascularity of many of the metastatic tumours. Theoretically, regional infusions of a chemotherapeutic agent will only increase drug concentrations to hypervascular areas in the liver. Clearly, therefore, administering a cytotoxic drug via the hepatic artery is inadequate to achieve a high tumour kill in hypovascular metastases and additional manipulation of the intrahepatic blood flow is required to improve drug delivery to the tumour. A variety of methods have been employed to alter blood flow to liver metastases. One such technique involves the use of degradable starch microspheres (DSM, Spherex, Pharmacia, Sweden) to induce temporary arterial embolisation. These microspheres, which have a mean diameter of 40 $\mu m$, consist of starch polymers cross-linked with epichlorhydrine. They are degraded by endogenous serum alpha-amylases and have a half-life in human serum of 25 min at 37°C *in vitro*. After regional administration via the hepatic artery the microspheres lodge in the small arterioles thereby slowing arterial inflow and resulting in intrahepatic arterial stasis. Regionally delivered cytotoxic drugs administered concomitantly with DSM are retained within the liver considerably longer than when the drugs are given alone (Lindell *et al.*, 1978; Lorelius *et al.*, 1984; Teder *et al.*, 1986). Furthermore, DSM preferentially enhances the delivery of drug to hepatic tumour (Chang *et al.*, 1989). However, it is unclear how these biodegradable emboli produce this effect. Therefore the aim of this study was to elucidate the underlying mechanisms of action of DSM in altering the intrahepatic distribution of a low molecular weight marker representing a cytotoxic drug. The experiments were designed to simulate as closely as possible the regional chemotherapy treatment of patients with colorectal liver metastases.

Correspondence: D Chang, Department of Surgery, Addenbrooke's Hospital, Cambridge CB2 2QQ, UK.
Received 26 January 1995; revised 23 November 1995; accepted 28 November 1995

### Materials and methods

### *Liver tumour model*

Liver tumours were established in hooded Lister rats (200-250 g body weight) by intraportal inoculation of a single-cell suspension of $5 \times 10^5$ HSN fibrosarcoma cells. This is a syngeneic cell-line grown from tissue culture. The rats were studied 21 days after administration of the tumorigenic cells when 4-8 discrete tumour nodules with a mean diameter of 7.5 mm were present in the liver of all the animals.

### *Tumour vascularity*

The vascularity of HSN sarcoma-derived tumour relative to the normal liver parenchyma was determined by histological examination and by the intrahepatic distribution of small radiolabelled, non-degradable $^{57}Co$ microspheres, 16.5 $\mu m$ in diameter (Nen-Trac, New England Nuclear, Stevenage, UK). After regional injection via the hepatic artery ($n=11$) or portal vein ($n=9$), these microspheres follow the paths of greatest flow and ultimately lodge at the intrahepatic presinusoidal level. Each injectate contained 40 000 microspheres which provided at least 400 microspheres per liver tissue sample, thereby minimising statistical errors (Buckberg *et al.*, 1971). In brief, 5 min after injection of the microspheres the rats were killed, the livers excised and divided into normal liver and tumour areas and the radioactivity of each section of liver measured in a gamma-well counter (1280 Ultragamma, LKB Wallac, UK). A schematic picture of the intrahepatic distribution of the microspheres was then built up and the ratio between tumour and normal liver tissue calculated.

*Degradable starch microspheres*

The serum amylase concentration of normal rats (range of 1000–1500 $Ul^{-1}$) is much higher than in man, and hence the starch microspheres used in human studies are degraded within seconds after intravascular administration in the rat. Therefore for our experiments microspheres were developed containing a greater number of cross-linkages (batch pH BR 58B43B 93769a), and a degradation time in the rat similar to that of human microspheres in man, i.e. a half-life of 25 min.

*Optimising the dose of DSM*

We have previously described a method for determining the optimal dose of starch microspheres to retain a low molecular weight marker within the liver using the Spherex monitoring system in a normal rat (Nott *et al.*, 1987). Applying the same technique to rats with hepatic tumours derived from HSN sarcoma cells we found that the dose of DSM which retained the maximum quantity of MDP within the liver without causing overspillage of microspheres into the splanchnic circulation was 2 mg.

*Low molecular weight marker*

The low molecular weight marker chosen for these experiments was $^{99}Tc^{m}$-labelled methylene diphosphonate (MDP). This marker is extensively used for bone scans and is not normally retained nor metabolised to any significant degree by the liver. However, MDP is of a similar size to commonly used cytotoxic drugs and is readily radiolabelled and counted. It has the additional advantages of rapid equilibration throughout the body, relative inexpense and is convenient to use.

*Procedures*

In all experiments the animals, fasted overnight but allowed access to water *ad libitum*, were anaesthetised with sodium pentobarbital (60 mg per kg body weight). Through a midline incision a 2 Fr Portex cannula was placed in the gastroduodenal artery such that its tip lay at the junction with the common hepatic artery. All subsequent injections given via this cannula were observed under direct vision using an operating microscope to ensure that the injection pressures did not produce backflow down the coeliac artery and hence into the splanchnic circulation. Blood pressure and heart rate were monitored continuously during each experiment by a pressure transducer (Medox Medical, Rossendale, UK) attached to a cannula placed in the left femoral artery. The volume of each injectate did not exceed 0.05 ml, this being the approximate arterial volume of the rat liver (Chang, 1993), and was administered using a high pressure liquid chromatography (HPLC) syringe. The low molecular weight marker (MDP) or $^{57}Co$ microspheres and DSM were thoroughly mixed with the aid of a vortex mixer immediately before administration.

*Control group*

A control group of 20 rats received a regional injection, via the hepatic artery, of MDP alone and the animals were killed, 1 min ($n = 10$) and 90 min ($n = 10$) later.

*Experimental groups*

In the first experimental group of 20 rats, MDP was co-injected with rat DSM (2 mg) via the hepatic artery. Ten animals were killed 1 min after injection and the remainder 90 min later. To compare the starch microspheres used in clinical studies a second group of 8 rats were given 2 mg of human DSM with MDP and killed after 90 min.

In the third series of experiments the hepatic artery or portal vein were occluded either before or after regional administration of the low molecular weight marker. In a group of eight rats immediately after injection of MDP the hepatic artery was clamped for 90 min and the animals then killed. In the next group of 16 rats the portal vein was clamped immediately before receiving an injection of MDP together with DSM. Eight rats were killed 1 min after injection, while in the other eight the clamp was removed after 1 min but the animals not killed for a further 90 min.

Freshly prepared MDP (30 $\mu g$ $mCi^{-1}$, with an activity ranging from 40 to 60 MBq $ml^{-1}$) and DSM (100 mg $ml^{-1}$) were used in each experiment. In all rats the livers were immediately excised and divided into normal liver and tumour areas resulting in 100–150 samples. Each sample, weighing between 100 and 250 mg, was placed at the bottom of a plastic vial and the radioactivity counted in a gamma-well counter. A schematic picture of MDP distribution in tumour and normal liver tissue could then be constructed. The activity of the injected dose of marker was determined by withdrawing and counting a volume equal to that injected in each experiment from the stock solution of MDP. In this way the concentration of marker in the tissues could be calculated in terms of percentage of the injected dose per milligram of the wet weight tissue (%ID per mg). The percentage of marker retained in tumour or liver for each group was calculated from the product of the total tissue weight and concentration of marker per mg of tissue.

A final group of 11 rats received co-injections of $^{57}Co$ microspheres and 2 mg of DSM, via the hepatic artery, to examine the effect of the starch microspheres on the intrahepatic distribution of the much smaller non-degradable microspheres. These animals were killed 5 min after injection, the livers excised and the activity counted in tumour and normal liver as previously described.

*Statistical analysis*

Results were analysed using non-parametric statistical methods. The Mann–Whitney *U*-test was used to assess significant differences between groups.

### Results

*Tumour vascularity*

For each group the mean weight of the tumours ranged from 20% to 26% of the total liver weight. Microscopic examination revealed irregularly shaped and densely packed cells with few vessels coursing through the tumour. The relatively hypovascular nature of this tumour was confirmed by the distribution of the $^{57}Co$ microspheres. Thus, following regional intra-arterial injection of $^{57}Co$ a greater number of microspheres was found in normal liver parenchyma than in tumour tissue resulting in a liver to tumour ratio of 2.25:1 (Table I). In addition, measurement of the lung counts demonstrated no significant arteriovenous shunting. Very few

**Table I** Intrahepatic radioactivity, median (range), after regional injection of $^{57}Co$ microspheres via the hepatic artery (HA) or portal vein (PV)

| | No. | Tissue radioactivity ($cps\ mg^{-1}$ tissue $\times 10^{-1}$) Normal liver (N) | Tumour (T) | N:T ratio | % arterio venous shunt |
|---|---|---|---|---|---|
| HA | 11 | 9.64 (4,92–13.01) | 4.28 (2.60–8.11) | 2.25 : 1 | 0.19 |
| PV | 9 | 9.50 (2.16–16.47) | 0.12 (0.03–0.44) | 79.17 : 1 | 1.15 |

microspheres reached the tumour from the portal circulation with 80 times more in normal liver compared with tumour tissue (Table I). Therefore, although the main blood supply to this tumour was from the hepatic artery the tumour blood flow was half that to the liver parenchyma.

*Intrahepatic marker distribution*

Figure 1 shows that 1 min after the intra-arterial injection of MDP alone there was a small but significantly ($P<0.02$) greater retention of marker in tumour [5.32% (2.74–6.72) of the injected dose per mg of tissue, median and range] compared with normal liver tissue (3.69, range 2.40–4.82). However, 1 min after the concomitant administration of DSM with MDP the retention of the marker within the liver was significantly increased compared to injection of marker alone ($P<0.001$) such that the total amount of marker retained in the liver increased from 5.81% to 23.76%. Moreover, the greatest concentration of MDP was found in tumour tissue (22.40, range 16.82–39.58) compared with normal liver parenchyma (9.08, range 4.85–12.59; $P<0.001$).

Ninety minutes after regional administration of the MDP the marker had equilibrated throughout the rat's body. In the control group of rats receiving MDP alone very little marker remained in the liver (1.27%) and there was no significant difference in the concentrations between tumour (0.77, range 0.55–1.43) and normal liver tissue (0.53, range 0.27–1.19). Co-injection of DSM with MDP did not affect the concentration of marker retained in normal liver tissue (0.61, range 0.28–1.40). In contrast 13 times more MDP was found in tumour (10.01, range 6.73–20.28; $P<0.001$) compared with that in the liver tumours of the control group despite the fact that at this stage over 95% of the starch microspheres had degraded. The total amount of marker retained within the liver was increased over five-fold to 7.12% of the injected dose.

A similar result was observed after 90 min when using the human DSM in the rat despite the more rapid degradation of these microspheres (Figure 2). In contrast, mechanical occlusion of the hepatic artery with a clamp had minimal effect on retention (1.54%) and intrahepatic distribution of the low molecular weight marker and there was no significant difference compared with the control group.

*Effects of portal venous occlusion*

The effects of manipulating portal venous inflow on the intrahepatic distribution of marker after co-injection with DSM are summarised in Figure 3. Occlusion of the portal vein produced intrahepatic venous stasis and increased the concentration of MDP in the liver. This effect was more pronounced in normal liver tissue (27.65, range 11.98–48.29), compared with tumour (8.65, range 5.72–17.99; $P<0.001$). Following the release of the venous clamp there was a significant reduction in these concentrations though by differing amounts for normal and tumour tissue. There was an 18-fold loss of marker from normal liver (1.56, range 0.51–4.36) compared with a 2-fold reduction in tumour (4.23, range 2.23–9.81; $P<0.001$). The total intrahepatic MDP concentration at 90 min was reduced to 4.64% compared with 7.12% during normal venous inflow largely as a result of the lower intratumour concentration. The concentration of marker washout from tumour and normal liver remained constant in all groups being 49% from tumour and 91% from liver tissue.

*DSM and $^{57}Co$ microspheres*

In the final experiment regional injection of DSM with $^{57}Co$ microspheres significantly altered the intrahepatic distribution pattern of the smaller radiolabelled microspheres. Compared with administration of the $^{57}Co$ microspheres alone the ratio of tumour to normal liver tissue was completely reversed (Figure 4). Furthermore, there was no change in the degree of arteriosystemic shunting.

**Figure 1** Concentration of MDP in the liver 1 min after regional injection of MDP alone or with DSM. Bars are medians.

**Figure 2** Concentration of MDP in the liver 90 min after regional injection of MDP alone, MDP and DSM, MDP and human DSM, and MDP alone but with the hepatic artery occluded. Bars are medians.

**Figure 3** Concentration of MDP in the liver after regional injection of MDP and DSM but with the portal vein occluded for 1 min. Bars are medians.

**Discussion**

Since the majority of liver metastases in man are isovascular or hypovascular (Healey, 1965; Kim *et al.*, 1977; Lin *et al.*, 1984), we developed an easily reproducible tumour model in the rat that reflects these characteristics. Direct observation of all the injections using an operating microscope allowed for the careful control of the animal's physiological parameters. Furthermore, by using DSM designed to degrade at the same rate as in man a more direct comparison can be made with clinical studies. Previously published

**Figure 4** Intrahepatic distribution of $^{57}$Co microspheres after regional injection alone or with 2 mg of DSM. Bars are medians.

animal work using these microspheres has not taken these factors into consideration.

In the control group of rats, given MDP alone via the hepatic artery, more marker initially accumulated in tumour than in the normal liver parenchyma. This was surprising in view of the comparatively poor arterial supply to the tumour but could result from different partition coefficients for the marker between the two tissues. However, substances injected into the hepatic artery can pass, via terminations of the arterial vessels into the portal vein and hepatic sinusoids (Rappaport, 1973; McCuskey, 1966), to the systemic circulation. Drugs and compounds like MDP which are not actively extracted by the liver will be washed out of the liver by this route with the portal venous inflow influencing the rate and volume of the washout. Thus the portal inflow may have rapidly washed out the marker from the normal liver but not from the tumour which has a poor portal venous supply. This is supported by the fact that 90 min after administration of MDP, when the marker has had sufficient time to equilibriate in the rat's body, there was no difference between the concentration of marker retained in normal liver and tumour.

Addition of DSM to the regional injectate of MDP immediately increased the intrahepatic concentration of marker. This initial increase in marker retention was expected from the DSM-induced vascular blockade and observed directly with the operating microscope. An unexpected finding was the selective localisation of MDP to the tumour. Two possible explanations may account for this phenomenon. First, since portal venous flow is unaffected by DSM arterial embolisation, less marker remains in normal liver than tumour owing to the portal venous washout, nullifying any stasis induced downstream to the DSM. Second, co-injection with DSM preferentially targets marker to tumour. The 3-fold difference in marker distribution between tumour and liver after concomitant administration with DSM suggests additional factors to portal washout are responsible for this effect. One such possibility is that DSM increases arterial blood flow to tumour thereby facilitating selective delivery of the marker.

When injected into the circulation microspheres tend to follow high flow pathways (Yipintsoi *et al*., 1973). Thus, the greatest degree of embolisation and hence marker entrapment, should occur within normal liver rather than the hypovascular HSN tumour. Paradoxically, in this study more MDP was retained within the tumour, and therefore the difference in the intrahepatic marker distribution cannot be explained merely on the basis of prolonged intra-arterial embolisation of DSM and sequestration of MDP. Furthermore, arterial stasis after clamping the hepatic artery *per se* did not significantly affect marker distribution. Therefore, in addition to producing intrahepatic arterial stasis DSM must alter intrahepatic blood flow thereby targeting the marker to tumour. Two factors indicate this was an immediate effect. First, the changes in marker distribution after injection of MDP with DSM were observed within the first minute. Second, co-administration of marker with human DSM, which are rapidly degraded in rats, resulted in a similar intrahepatic distribution of MDP at 90 min as that observed with the specifically designed rat DSM. Moreover, this accounts for the results of previous studies which have reported increased drug concentrations in experimental tumour 5 min (Flowerdew *et al*., 1987) or 30 min (Sigurdson *et al*., 1986) after co-injection with human DSM.

In the present study, 90 min after regional injection of MDP and DSM, when over 95% of the starch microspheres had degraded, the concentration of marker retained in normal liver was comparable with the control group receiving MDP alone. In contrast 13 times more MDP remained in tumour compared with the control group. Thus the disparity in marker distribution was not only maintained after DSM degradation but was amplified, possibly owing to a combined effect of DSM-induced tumour arterial stasis and relatively poor portal washout of marker from tumour.

As expected, reduction of the portal venous inflow by occlusion of the portal vein further increases the concentration of marker within the liver. However, despite the presence of DSM, relatively more marker was now localised in normal liver and less in tumour compared with the group whose portal flow was uninterrupted, even after restoration of portal flow and degradation of the DSM. It is known that a reduction in portal venous flow leads to a compensatory increase in hepatic arterial flow (Greenway and Oshiro, 1972). Furthermore, Ackerman *et al*. (1972) reported that acute ligation of the portal vein in rats with overt liver tumours diverts blood flow from tumour to liver. They suggested that this was caused either by better arterial perfusion to liver than to tumour or the shunting of arterial blood to the intrahepatic portal circulation. In the present study the increase in hepatic arterial flow secondary to the reduced portal venous flow in animals with HSN tumour will be proportionally greater in normal liver than the less vascular tumour, which may account for the relatively greater accumulation of DSM and co-injected marker in liver tissue. These results provide further evidence that the intrahepatic vascular flow pattern largely determines the distribution of the DSM. In addition, the results suggest that interference with the portal blood flow will have a deleterious therapeutic effect on intra-arterially injected cytotoxic drugs given with DSM.

The results from the final part of this study clearly indicate that when regionally co-injected with DSM, significantly more of the radiolabelled $^{57}$Co microspheres were entrapped in tumour compared with the normal liver. The reversed tumour to liver ratio for $^{57}$Co activity after injection with DSM suggests a 4.5-fold increase in arterial blood flow to tumour which corresponds with the initial 4-fold increase in the accumulation of MDP in tumour after co-injection with DSM. Changes in liver tumour vascularity after DSM embolisation have also been demonstrated in patients using high-flow dynamic CT scanning (Civalleri *et al*., 1985). These authors proposed that the arteriolar–capillary blockade induced by DSM produces an increased arterial back pressure that forcibly redistributes blood flow within the liver. Alternatively local DSM embolisation may open up arterio–arteriovenous shunts hitherto temporarily closed (McCuskey, 1966). The intrahepatic shunting of blood may be mediated via a temporary hypoxia induced by DSM (Arfors *et al*., 1976) or reduction in the local washout of adenosine (Lautt, 1985). As the DSM obstruct flow in one channel these 'new' pathways allow the passage of co-injected drugs to initially relatively ischaemic areas in the liver.

The selective targeting of a low molecular weight marker to a hypovascular hepatic tumour after co-injection with DSM can therefore be explained by an initial blockade of hypervascular areas within the liver by microspheres with a redistribution of flow to less vascular areas. The marker then remains in tumour for longer than in the liver parenchyma due to a combination of poor portal washout from tumour and interruption of the tumour's main blood supply by DSM. This effect will increase the local marker concentration and encourage tumour uptake of marker.

Concomitant administration of DSM and cytotoxic drugs which, unlike MDP, are actively retained by the liver may result in even higher concentrations within liver tumours. Furthermore, DSM could enhance the effects of internal radiotherapy for liver metastases using regionally injected yttrium-90-labelled microspheres (Burton *et al.*, 1989). Addition of the larger DSM may target the radioactive microspheres to hypovascular tumours thereby sparing normal hepatocytes. These results warrant further investigation into the clinical effects of DSM on the targeting of cytotoxic agents to hypovascular liver tumours.

**Acknowledgements**
This study was supported by the Cancer Research Campaign and North West Cancer Research Fund. We would like to thank Dr SA Eccles, Institute of Cancer Research, Sutton, Surrey, UK, for donating the tumour cell line and Pharmacia Ltd, Sweden, for supplying the DSM. We are indebted to Mr J Yates for his technical assistance.

### References

ACKERMAN NB. (1974). The blood supply of experimental liver metastases IV. Changes in vascularity with increasing tumour growth. *Surgery*, **75**, 589–596.

ACKERMAN NB, LIEN WM AND SILVERMAN NA. (1972). The blood supply of experimental liver metastases. III the effects of acute ligation of the hepatic artery and portal vein. *Surgery*, **71**, 636–641.

ARFORS KE, FORSBERG JO, LARSSON B, LEWIS DH, ROSENGREN B AND ODMAN S. (1976). Temporary intestinal hypoxia induced by degradable microspheres. *Nature*, **262**, 500–501.

BUCKBERG GD, LUCK JC, PAYNE DB, HOFFMAN JIE, ARCHIE JP AND FIXLER DE. (1971). Some sources of error in measuring regional blood flow with radioactive microspheres. *J. Appl. Physiol.*, **31**, 598–604.

BURTON MA, GRAY BN, KLEMP PF, KELLEHER DK AND HARDY N. (1989). Selective internal radiation therapy: distribution of radiation in the liver. *Eur. J. Cancer Clin. Oncol.*, **25**, 1487–1491.

CHANG D. (1993). Targeting regional chemotherapy to hypovascular liver tumours: the role of degradable starch microspheres. MD thesis. University of Manchester.

CHANG D, JENKINS SA, NOTT DM, GRIME S AND COOKE T. (1989). Biodegradable emboli increase the delivery of cytotoxic drug to hepatic tumour. *Br. J. Surg.*, **76**, 631.

CIVALLERI D, ROLLANDI G, SIMONI GA, MALLARINI G, REPETTO M AND BONALUMI U. (1985). Redistribution of arterial blood flow in metastases-bearing livers after infusion of degradable starch microspheres. *Acta Chir. Scand.*, **151**, 613–617.

ENSMINGER WD AND GYVES JW. (1983). Clinical pharmacology of hepatic arterial chemotherapy. *Semin. Oncol.*, **10**, 176–182.

FISHER ER AND TURNBULL RB. (1955). The cytologic demonstration and significance of tumour cells in the mesenteric venous blood in patients with colorectal carcinoma. *Surg. Gynecol. Obstet.*, **100**, 102–108.

FLOWERDEW ADS, RICHARDS HK AND TAYLOR I. (1987). Temporary blood flow stasis with degradable starch microspheres for liver metastases in a rat model. *Gut*, **28**, 1201–1207.

GREENWAY CF AND OSHIRO G. (1972). Comparison of the effects of hepatic nerve stimulation on arterial flow, distribution of arterial and portal flow and blood content in the livers of anaesthetized cats and dogs. *J. Physiol.*, **227**, 487–501.

HEALEY JE. (1965). Vascular patterns in human metastatic liver tumors. *Surg. Gynecol. Obstet.*, **120**, 1187–1193.

KIM DK, WATSON RC, PAHNKE LD AND FORTNER JG. (1977). Tumor vascularity as a prognostic factor for hepatic tumors. *Ann. Surg.*, **185**, 31–34.

LAUTT WW. (1985). Mechanism and role of intrinsic regulation of hepatic arterial blood flow: the hepatic arterial blood flow response. *Am. J. Physiol.*, **249**, G549–556.

LIN G, LUNDERQVIST A, HAGERSTRAND I AND BOIJSEN E. (1984). Postmortem examination of the blood supply and vascular pattern of small liver metastases in man. *Surgery*, **96**, 517–526.

LINDELL B, ARONSEN K-F, NOSSLIN B AND ROTHMAN U. (1978). Studies in pharmacokinetics and tolerance of substances temporarily retained in the liver by microsphere embolization. *Ann. Surg.* **187**, 95–99.

LORELIUS LE, BENEDETTO AR, BLUMHARDT R, GASKILL HV, LANCASTER JL AND STRIDBECK H. (1984). Enhanced drug retention in VX2 tumors by use of degradable starch microspheres. *Invest. Radiol.*, **19**, 212–215.

MCCUSKEY RS. (1966). A dynamic and static study of hepatic arterioles and hepatic sphincters. *Am. J. Anat.*, **119**, 455–477.

NEIDERHUBER JE, ENSMINGER W, GYVES J, THRALL J, WALKER S AND COZZI E. (1984). Regional chemotherapy of colorectal cancer metastatic to the liver. *Cancer*, **53**, 1336–1343.

NOTT DM, YATES J, GRIME JS, MALTBY P, O'DRISCOLL PM, BAXTER JN, JENKINS SA AND COOKE TG. (1987). Induced hepatic arterial blockade by degradable starch microspheres in the rat. *Nucl. Med. Commun.*, **8**, 1019–1025.

RAPPAPORT AM. (1973). The microcirculatory hepatic unit. *Microvasc. Res.*, **6**, 221–228.

SCHWARTZ SI, JONES LS AND MCCUNE CS. (1985). Assessment of treatment of intrahepatic malignancies using chemotherapy via an implantable pump. *Ann. Surg.*, **201**, 560–567.

SIGURDSON ER, RIDGE JA AND DALY JM. (1987). Intra arterial infusion of Doxorubicin with degradable starch microspheres. Improvement of hepatic tumor drug uptake. *Arch. Surg.*, **121**, 1277–1281.

TEDER H, ARONSEN KF, BJORKMAN S, LINDELL B AND LJUNGBERG B, (1986). The influence of degradable starch microspheres on liver uptake of 5-fluorouracil after hepatic artery injection in the rat. *J. Pharm. Pharmacol.*, **38**, 939–941.

YIPINTSOI T, DOBBS JR WA, SCANLON PD, KNOPP TJ AND BASSINGTHWAIGHTE JB. (1973). Regional distribution of diffusible tracers and carbonized microspheres in the left ventricle of isolated dog hearts. *Circ. Res.*, **33**, 573–587.